# Inflammatory-Targeted Lipid Carrier as a New Nanomaterial to Formulate an Inhaled Drug Delivery System

**DOI:** 10.3390/molecules29071616

**Published:** 2024-04-03

**Authors:** Eleonora Maretti, Federica Gioia, Cecilia Rustichelli, Susanna Molinari, Eliana Leo

**Affiliations:** Department of Life Sciences, University of Modena and Reggio Emilia, via G. Campi 103, 41125 Modena, Italy; eleonora.maretti@unimore.it (E.M.); federica.gioia96@gmail.com (F.G.); cecilia.rustichelli@unimore.it (C.R.); susanna.molinari@unimore.it (S.M.)

**Keywords:** Palmitoylethanolamide, PEA, nanoparticles, nanocarrier, pulmonary administration, lung disease, aerosol, respirability, DPI, dry powder

## Abstract

There is a pressing need for efficacious therapies in the field of respiratory diseases and infections. Lipid nanocarriers, administered through aerosols, represent a promising tool for maximizing therapeutic concentration in targeted cells and minimizing systemic exposure. However, this approach requires the application of efficient and safe nanomaterials. Palmitoylethanolamide (PEA), an endocannabinoid-like endogenous lipid, plays a crucial role in providing protective mechanisms during inflammation, making it an interesting material for preparing inhalable lipid nanoparticles (LNPs). This report aims to preliminarily explore the in vitro behavior of LNPs prepared with PEA (PEA-LNPs), a new inhalable inflammatory-targeted nanoparticulate drug carrier. PEA-LNPs exhibited a size of about 250 nm, a rounded shape, and an marked improvement in PEA solubility in comparison to naked PEA, indicative of easily disassembled nanoparticles. A twin glass impinger instrument was used to screen the aerosol performance of PEA-LNP powders, obtained via freeze-drying in the presence of two quantities of mannose as a cryoprotectant. Results indicated that a higher amount of mannose improved the emitted dose (ED), and in particular, the fine particle fraction (FPF). A cytotoxicity assay was performed and indicated that PEA-LNPs are not toxic towards the MH-S alveolar macrophage cell line up to concentrations of 0.64 mg/mL, and using coumarin-6 labelled particles, a rapid internalization into the macrophage was confirmed. This study demonstrates that PEA could represent a suitable material for preparing inhalable lipid nanocarrier-based dry powders, which signify a promising tool for the transport of drugs employed to treat respiratory diseases and infections.

## 1. Introduction

Many lung diseases involve chronic inflammation, a natural response to injuries caused by various factors, such as pathogens, toxins, irritants, and allergens. Excessive inflammation can lead to conditions like chronic obstructive pulmonary disease (COPD) and asthma [1,2]. Administering drugs via the pulmonary route enables local delivery of therapeutics, avoiding the requirement for therapeutic injections. The advantages of the pulmonary route include higher rates of drug absorption and lower required drug doses compared to conventional systemic administration [3]. However, low retention of drugs in the lungs may occur due to the distinctive physiological barriers present. Therefore, traditional inhaled administration methods often encounter limitations that require innovative approaches to overcome [4,5]. Enhancing the therapeutic impact of drugs for pulmonary injuries via the use of efficient drug delivery systems poses a persistent challenge in the field.

Among the strategies to improve pulmonary drug delivery, lipid nanoparticle-based dry powders have demonstrated promising potential as next-generation respiratory medicines [6]. This is for two primary reasons: (i) they serve as drug carriers that can overcome mucociliary clearance requirements, since nanoparticles can effectively cross the mucus barrier, leading to prolonged retention at the cell surface; and (ii) they are highly advantageous in enhancing the targeting and uptake of therapeutics by alveolar macrophages. Indeed, macrophages play an important role in the pathogenesis process, as they are involved in innate immunity and are responsible for the initiation, maintenance, and resolution of inflammation. Phagocytosis, the principal activity of macrophages, presents a dual impact on drug delivery through inhalation. On one hand, it significantly contributes to drug elimination, thereby reducing effective drug concentrations at the intended site [7]. Conversely, in infectious diseases, pathogenic microorganisms (i.e., Mycobacterium tuberculosis) reside and replicate within alveolar macrophages, and phagocytosis becomes crucial as macrophages serve as the drug target [3]. The approval of Arikayce^®^ (Insmed Inc., Bridgewater, NJ, USA) to treat non-tuberculous mycobacteria infections solidified the potential of antibiotics packaged into lipid nanoparticles for localized lung delivery [8,9]. Although nano-formulations have great potential, they still pose many unknown risks [10]. For instance, a large body of evidence suggests that some nanoparticles can accumulate in the lungs after inhalation and induce lung nodules or even tumors [11,12]. Therefore, the use of matrices with low toxicity and that do not easily accumulate is an essential prerequisite.

The anti-inflammatory effects of Palmitoylethanolamide (PEA), an endocannabinoid-like lipid belonging to the family of *N*-acylethanolamines, suggest a potential role in mitigating lung diseases and infections. PEA is an endogenous lipid involved in spontaneous protective mechanisms, which are triggered by tissue damage or inflammatory responses. It is not stored in the body; it is synthesized as needed through a two-step process, involving the formation of *N*-acyl-phosphatidylethanolamine (NAPE) and the subsequent release of PEA by an enzyme called NAPE-PLD [13]. PEA may bind various molecular targets, including PPAR-α and GPR55, and it indirectly affects cannabinoid receptors and the TRPV1 receptor via PPAR-α, leading to the inhibition of expression of pro-inflammatory enzymes and the reduction of nitric oxide and pro-inflammatory cytokines. Early results from animal studies supported the idea that PEA was a non-specific enhancer of host defenses against bacterial and viral infection, while at the same time exerting anti-inflammatory activity [14]. Recently, an ultra-micronized form of PEA has been proposed as an adjuvant treatment for COVID-19, with the potential to reduce inflammation, oxidative stress, and coagulation issues in patients [15]. Further studies have shown that adelmidrol (a PEA analogue) can reduce inflammation and airway hyperreactivity in asthma, and lower inflammatory markers in idiopathic pulmonary fibrosis [16]. This suggests that PEA is a safe lipid with potential intrinsic anti-inflammatory activity that has not yet been used as excipient for lipid nanoparticle formulation. The application of PEA as a matrix material (lipid) in the formulation of nanocarriers would address the need for systems with better bioactivity and biosafety, and represents, to our knowledge, a novelty in the landscape of lipid carrier formulation.

Taking into account the provided context, this report delves into the development of a novel inhalable lipid nano-drug delivery system which exhibits intrinsic anti-inflammatory activity, thus bringing innovation to the panorama of carriers for aerosol therapy. These novel inflammatory-targeted lipid nanoparticles were formulated using PEA as a lipid (PEA-LNPs). The characterization of PEA-LNPs involved an assessment of their size, zeta potential, and morphology. The developed PEA-LNPs were subjected to freeze-drying and analyzed by a twin glass impinger (TGI) to screen their potential for inhaled therapy. Furthermore, their cytotoxicity and extent of cellular internalization were evaluated using the MH-S alveolar macrophage cell line.

## 2. Results and Discussion

### 2.1. PEA-LNPs Preparation and Characterization

In the ever-evolving landscape of combating respiratory infectious diseases and viral threats, the development of new particulate carriers that not only reduce the potential side effects of drugs, but also possess inherent anti-inflammatory properties, is challenging.

Preliminary studies using varying quantities of PEA and stearic acid to produce novel LNPs were carried out. Some of the formulations obtained with different amounts of PEA are detailed, along with their dimensional characterization, in the supplementary material (Table S1). The formulation studied here represents the final formulation with the best characteristics in terms of size and polydispersity index.

Standardized PEA-LNPs showed a size of 253 ± 15 nm with PDI of 0.185 ± 0.100, indicating the presence of a single particle population. Homogenous particle size is a key parameter for stability, biodistribution, drug release, and cellular uptake [17]. Involved particles exhibited a negative surface charge value (−39.5 ± 2.1 mV), in agreement with the presence of stearic acid in the composition. The negatively charged surface may promote macrophage uptake and reduce possible local inflammation after administration, in comparison with cationic charge particles [18,19].

**Figure 1:**
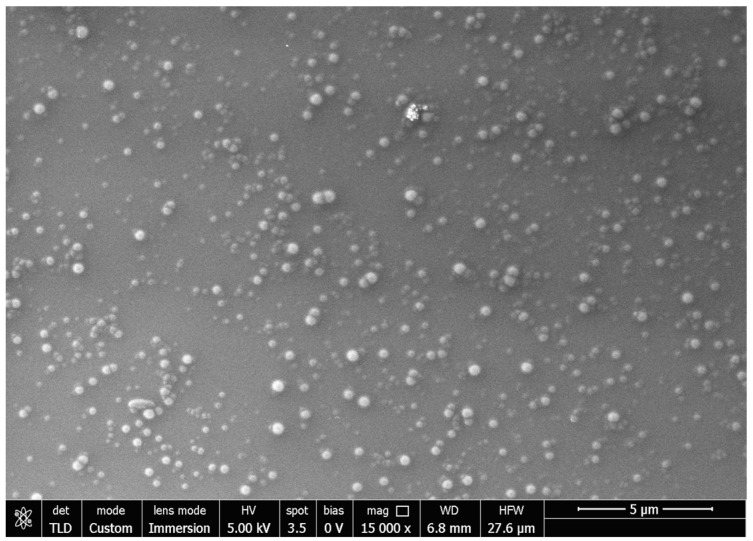
SEM image of PEA-LNPs.

With the aim of confirming the dimensional analysis, PEA-LNPs were evaluated morphologically using SEM. Particles showed a spherical shape, and their size was almost in agreement with the results obtained by the light scattering analysis (Figure 1).

### 2.2. In Vitro PEA Dissolution Rate

**Figure 2:**
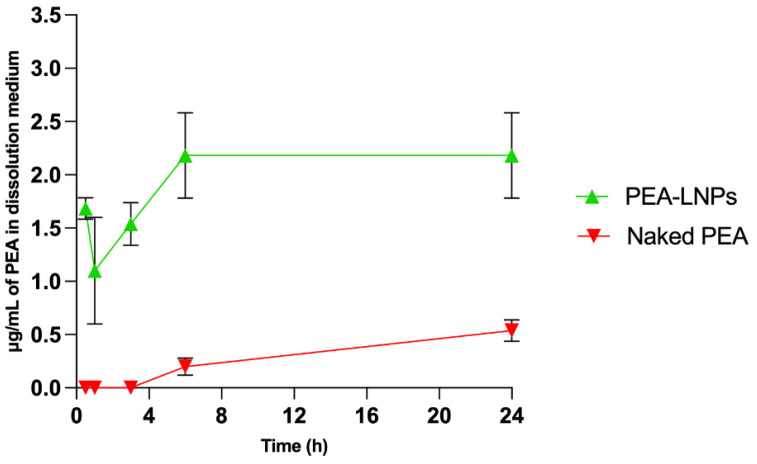
Dissolution of naked PEA in comparison to PEA-LNPs in simulated lung fluid (SLF) at pH 7.4. Results are presented as the average ± SD (*n* = 3).

The determination of the in vitro PEA dissolution rate from LNPs was carried out using a simulated lung fluid (SLF) at pH 7.4 and evaluated in comparison with the dissolution rate of naked PEA. To assess the effective difference in dissolution rate between naked PEA and PEA-LNPs, the test was carried out in a large volume of SLF (250 mL); however, this is not comparable to the amount of fluid present at lung level, owing to the very low solubility of PEA in water (<0.1 μg/mL). As shown in Figure 2, the difference in dissolution rate between the two forms of PEA is particularly marked (*p* < 0.01). In fact, in the experimental conditions adopted, after 24 h, naked PEA reached a concentration of 0.5 μg/mL, while PEA-LNPs reached a concentration of about five times higher (2.5 μg/mL). The elevated initial dissolution of PEA from LNPs can be explained by its amorphization during matrix formation along with stearic acid, as observed in a previous paper [20]. This may induce a rapid erosion of the carrier and consequently a release of the incapsulated drug.

### 2.3. Freeze-Dry Powder Characterization and In Vitro Respirability

With the aim of using PEA-LNPs via the inhalation route, nanoparticle suspensions were freeze-dried under pre-selected conditions [21]. As can be observed in Table 1 and Table 2, in the absence of cryoprotectants, freeze-dried PEA-LNPs produced a powder with worse flowability and respirability characteristics than lipid nanoparticles produced in the absence of PEA (C-LNPs). The worsening of these properties is probably due to PEA, as the presence of certain lipids could make the powder sticky, forming a bridge between particles and reducing the flowability [22].

For this reason, the addition of a cryoprotectant was mandatory. In particular, mannitol, one of the most widely used cryoprotectants, was added before the freeze-drying process at 1:2 and 1:1 lipid/cryoprotectant ratios.

Density, angle of repose, Housner ratio, and Carr’s index for freeze-dried PEA-LNPs were evaluated and are reported in Table 1.

The void fraction and density are the most important properties of particulate inhalable materials, as well as aerodynamic size, shape, surface morphology, and porosity. All of these are critical in order to achieve adequate powder processability, desired lung deposition, and enhanced bioavailability. Due to their low density (<0.4 g/cm^3^), porous particles with a geometric diameter >5 μm can be delivered into the deep lung [23]. The packing or tap density of a powder depends on how close the particles are able to be packed in a powder bed, which is related to the cohesive forces between particles. PEA-LNPs freeze-dried with mannitol exhibit similar values of bulk density (0.04–0.05 g/cm^3^) and tap density (0.05 g/cm^3^), suggesting high flowability of the powder, owing to the presence of poor inter-particle spaces removed with packing. Moreover, both the measured density values are very low, indicating the suitability of the powder for inhaled administration. In particular, tap density is a fundamental parameter for flowability, and it is known that particles with a low tapped density can be aerosolized more efficiently through a DPI device [24,25]. It is worth noting that the density values obtained with the cryoprotectant are lower than those obtained without it, suggesting that the cryoprotectant exerts an important action in improving the flowability of the powder. Powder flow properties were also evaluated through the determination of Hausner ratio, Carr’s Index, and the angle of repose (Table 1). According to Ph.Eur. [26], Hausner ratio and Carr’s Index are two parameters theoretically calculated using the densities [27,28], while the angle of repose is a parameter related to the inter-particle spaces and expresses the resistance that may exist between the particles [29]. Among the samples analyzed, when comparing the three parameters with the values reported in the reference tables provided by the Pharmacopoeia [26], PEA-LNPs without mannitol fell within the ‘poor’ range; PEA-LNPs at a 1:1 ratio exhibited a slight improvement, placing them in the ‘fair’ range; and the most favorable performance was observed in PEA-LNPs at a 1:2 lipid/mannitol ratio, which demonstrated all three parameters within the ‘excellent’ range. The addition of a significant amount of mannitol contributes to enhancing flow characteristics, potentially leading to improved emitted dose from the DPI device. The bad flowability performances of PEA-LNPs without mannitol could be attributable to the presence of high Van der Waals forces between particles [30]. On the other hand, the role of mannitol as a cryoprotectant in lyophilized protein formulations, where it also serves as a bulking agent, is well known [31]. Indeed, mannitol might modify the surfaces of the lipid particles, reducing their inherent waxy-like cohesive effects.

To better understand the ability of powder to be inhaled, a preliminary test was performed using the twin glass impinger (TGI) apparatus (as described in European Pharmacopoeia [32]), suitable for an initial in vitro screening of respirability performance. The obtained results were expressed as emitted dose (ED) percentage values, representing the quantity of PEA-LNPs fully released by the DPI device. Additionally, the respirable fraction was divided into two phases: the fine particle fraction (FPF), indicating the proportion of particles with a size less than 5 µm, capable of reaching the deepest regions of the lungs, and the large particle fraction (LPF), corresponding to particles larger than 5 µm, which tend to settle in the upper or central airways (Table 2).

A favorable test outcome was obtained when the total recovered powder percentage was higher than 80% for all of the samples, considering that Ph.Eur. specifies a valid range between 75 and 125% [32]. In the case of PEA-LNPs without mannitol, both the ED and respirable fraction values were notably low, highlighting the powder’s unsuitability for inhalation administration. Specifically, the FPF stood at approximately 0.1%, while the more critical ED metric indicated that only 60% of the powder could be expelled from the DPI device (Table 2). Regarding the two PEA-LNPs prepared with cryoprotectants, their similar ED values of approximately 80% classify them as suitable preparations for administration via the inhalation route. On the other hand, a significant difference (*p* < 0.05) in the respirable fraction, notably the FPF, was observed between samples prepared with different amounts of mannitol. In particular, the two PEA-LNPs exhibited FPF values of 9.6 ± 3.2% and 16.4 ± 1.7% for 1:1 and 1:2 lipid/cryoprotectant ratios, respectively. This underscores that a higher quantity of cryoprotectant promotes the formation of a more respirable powder with the potential to reach deeper airways. It is well-documented that particles with a low angle of repose can be aerosolized more efficiently through a DPI device, resulting in a higher FPF [33]. However, a further increase in the amount of cryoprotectant was not considered as an option, because a high excess of bulk agent is not desirable in the treatment of inflammatory airway diseases.

### 2.4. In Vitro Studies on Macrophage MH-S Cell Line

The cytotoxicity of PEA-LNPs was evaluated via MTT assay on the MH-S cell line after 6 h of incubation using three different concentrations, 0.16, 0.32, and 0.64 mg/mL, of both PEA-LNPs and C-LNPs.

**Figure 3:**
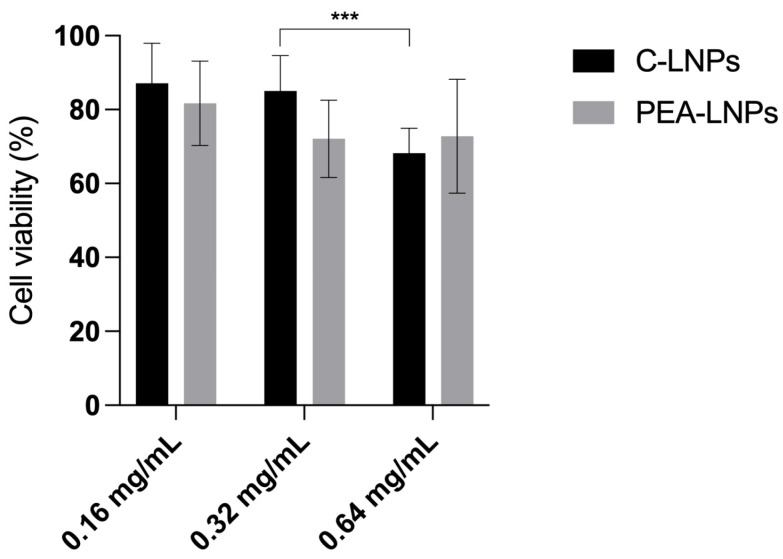
Cell viability after 6 h of treatment with C-LNPs and PEA-LNPs at three different concentrations. Statistical significance levels are indicated as *** (*p* < 0.01).

C-LNPs exhibited a typically dose-dependent cytotoxicity, with similar values at lower concentrations (0.16 and 0.32 mg/mL), and a significative (*p* < 0.01) cell viability reduction at 0.64 mg/mL, indicating a certain toxicity of the carrier (Figure 3). The toxicity of lipid carriers with different compositions has been widely studied in different cell lines, and in most cases, a dose-dependent toxicity was found [34].

PEA-LNPs were not found to behave in the same way. Indeed, among the three concentrations, no significative differences were observed (*p* > 0.05) (Figure 3). As PEA is a lipid with low toxicity [35] that is very well-tolerated by cells, it can be hypothesized that its presence in the nanoparticles composition may mitigate the inherent toxicity of the nanoparticles, making PEA-LNPs a carrier with high exploitation potential in the panorama of drug delivery systems.

The internalization assay was performed via labelling PEA-LNPs (0.32 mg/mL) with coumarin-6 dye and observing cells through flow cytometry and confocal microscopy, using a corresponding amount of unlabeled C-LNPs and untreated cells as negative controls. Before the analysis, the stability of the marker (coumarin-6) in the PEA-LNPs was evaluated for 24 h, as described in the ‘Materials and Methods’ section. During this time period, the dye coumarin-6 was not released at all from the labelled PEA-LNPs, meaning the marker was stably associated to the carrier.

In order to quantify the extent of internalization, flow cytometry analysis was conducted only at 1 and 3 h, since at 6 h particle digestion starts, possibly leading to inaccurate results [36].

No intrinsic fluorescence was observed for untreated cells and cells treated with unlabeled C-LNPs (Table 3).

Per evaluation of the mean fluorescence intensity of coumarin-6, the labelled PEA-LNPs were largely taken up by lung macrophages after 1 h of incubation (*p* < 0.01). Indeed, a very high percentage (94%) of positive fluorescent cells was detected. The overall level of uptake of PEA-LNPs was maintained or slightly increased after 3 h.

**Figure 4:**
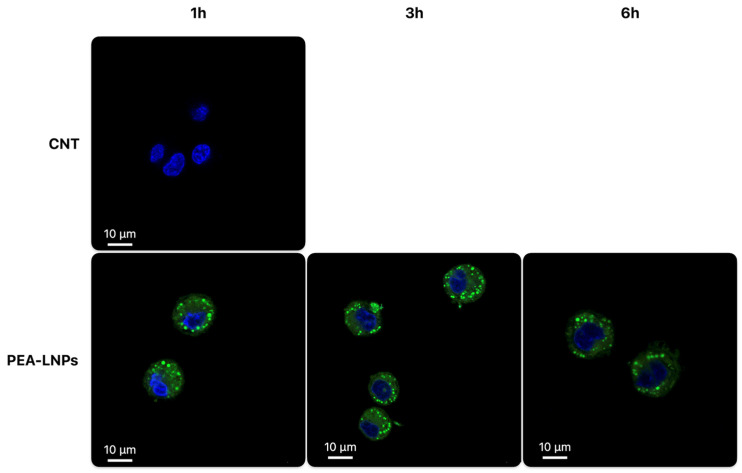
Confocal microscopy images of MH-S cells treated with coumarin-6 labelled PEA-SLNs after 1 h, 3 h, and 6 h, in comparison with not treated cells (CNT).

In order to verify whether the particles were in the cytoplasm and not on the membrane of cells, a confocal microscope analysis was conducted only on cells treated with coumarin-6 labelled PEA-LNPs. The confocal analysis was conducted also after 6 h of incubation time (Figure 4) in order to observe the cells even upon their possible initial degradation. After 1 h of incubation, colorful spots were visible inside the cell cytoplasm, indicating a rapid internalization process. These spots probably represented LNPs aggregated inside cytoplasmatic vesicles such as lysosomes or phagosomes. After 3 h, no substantial differences with respect to 1 h were observed, while after 6 h, a formation of black spaces inside the cells began to be visible, probably attributable to the late endosomes, indicative of the digestion and destruction of the particles. After this time period, presumably, the dissolution of PEA, according to the dissolution test results, also occurred.

The confocal analysis thus confirmed the data obtained with the flow cytometer, highlighting a massive and rapid internalization of PEA-LNP particles into MH-S cells. The large macrophage uptake exhibited by PEA-LNPs could be of great benefit in the use of the carrier in inhaled therapy for the delivery of drugs to treat disseminated infections, such as mycobacterium or visceral leishmaniasis affecting the lungs [37,38,39]. This behavior is closely in accordance with that observed in the literature for several kinds of lipid carriers (SLN, NLC, and liposomes) used on different cell lines [40,41,42]; therefore, this in itself does not represent an innovative result. However, it is important to underline that PEA as an excipient can constitute a nanocarrier with inherent anti-inflammatory activity, in addition to its potential ability to encapsulate anti-infective drugs. In fact, it has been extensively demonstrated that PEA modulates the expression of enzymes involved in pro-inflammatory processes, such as Cyclooxygenase-2 (COX-2) and nitric oxide synthase (iNOS), and reduces pro-inflammatory cytokine production, mainly via up-regulation of the nuclear receptor PPAR-α [43,44]. Moreover, PEA appears to be able to stimulate macrophages, even those already activated, to increase their phagocytic activity against pathogens such as Streptococcus pneumoniae, reducing their intracellular survival [45,46].

Therefore, this carrier could be used to transport anti-infective drugs to macrophages to target the etiological agent at the site of replication, while at the same time, it could also promote pathogen phagocytosis and exert anti-inflammatory action, reducing and alleviating the symptoms associated with infectious diseases.

## 3. Materials and Methods

### 3.1. Materials

PEA (Opti-PEA) was supplied by Innexus Nutraceuticals (Nijmegen, The Netherlands). Stearic acid, cholesteryl stearate, Span 85, Pluronic F68, and mannitol were purchased from Sigma-Aldrich Co. (St. Louis, MO, USA). Coumarin-6 was purchased from Acros Organics (Bridgewater, NJ, USA).

Simulated lung fluid type 3 (SLF) at pH 7.4, used for in vitro drug dissolution, was prepared according to the method described by Marques [47], as reported in the Supplementary Materials (Table S2).

For the cytotoxicity and cell internalization investigations, an MH-S cell line from IZSLER (Brescia, Italy), cell culture reagents RPMI-1640 medium, and phosphate-buffered saline (PBS) were purchased from Sigma-Aldrich (St. Louis, MO, USA). Trypsin EDTA and L-Glutamine from Lonza (Bornem, Belgium), penicillin-streptomycin (P/S) from Cambrex Bio Science Verviers (Liège, Belgium), fetal bovine serum (FBS) from PAN-Biotech (Aidenbach, Germany), paraformaldehyde from Sigma-Aldrich (St. Louis, MO, USA), Hoechst 33,342 from ThermoFisher (Monza, Italy), and thiazolyl blue tetrazolium bromide (MTT) from Panreac Applichem ITW Reagents (Milan, Italy) were employed.

All the other chemicals were of analytical grade.

### 3.2. Lipid Nanoparticles Preparation

PEA based lipid nanoparticles (PEA-LNPs) were prepared using the melt emulsification technique [20]. For the standardized formulation, a blend of PEA, stearic acid, and cholesteryl stearate (1:2.6:2 weight ratio) containing Span 85 (30%, *w*/*w*) was melted at a temperature of 85 °C, 10 °C above the melting point of the lipid. Then, the aqueous phase (5 mL Milli-Q water) containing 0.3% Pluronic F68 was heated at the same temperature and added to the lipid phase. Emulsification was performed via ultrasounds (SFX150 Branson, Milan, Italy) for 1 min, followed by homogenization using Ultra-Turrax (T-25 basic, Ika Labortechnik, Germany) at 24,000 rpm for 1.5 min and sonication for 1 min. The obtained oil-in-water emulsion was cooled in an ice bath under magnetic stirring for 15 min to facilitate the solidification of LNPs, and then purified by dialysis membrane (MWCO 12–14,000 Da) for 1 h in 300 mL Milli-Q water. After dialysis, the volume of suspension was adjusted to 5 mL and used for further analysis. Conventional LNPs (C-LNPs) were obtained using the same method, only without PEA.

### 3.3. Morphology and Size

The morphology of standardized PEA-LNPs was observed using scanning electron microscopy (SEM, Nova NanoSEM 450, Fei, Eindhoven, The Netherlands) with a TDL detector. Particle suspension was dropped on an aluminum stub, and after drying, coated with carbon under vacuum conditions (Carbon Coater, Balzers CED-010, Oerlikon Balzers, Balzers, Liechtenstein).

Size, polydispersity index (PDI), and surface charge (Z-potential) were measured by light scattering using Zetasizer PRO—Red Label (Malvern Panalytical, Worcs, UK) equipped with a 10 mW He-Ne laser (632.8 nm) and ZS Xplorer software (version n. 3.1.0.64).

### 3.4. PEA Dissolution Rate

PEA dissolution was determined in simulated lung fluid (SLF) at pH 7.4. About 17 mg of PEA-LNP was incubated in 250 mL of SLF under magnetic stirring at 37 ± 1 °C, in comparison with a similar amount of naked PEA. At fixed time intervals (30 min, 1 h, 3 h, 6 h, and 24 h) an aliquot of the suspension (1 mL) was withdrawn and subjected to centrifugation at 6000× *g* for 30 min (Rotina 380R, Hettich, Germany) in 100 kDa MWCO Vivaspin columns (Sartorius, Goettingen, Germany). After each withdrawal, 1 mL of fresh dissolution medium was added to maintain a constant volume. PEA concentration in the purified aliquots was determined by HPLC analysis, as described below.

### 3.5. HPLC Analysis

Analyses were carried out using a JASCO high-performance liquid chromatograph (Jasco Corporation, Tokyo, Japan) equipped with two PU-2080 Plus pumps, an HG-980-30 solvent mixing module, and a UV-2075 Plus UV-vis detector. Manual injection was performed by a Rheodyne 7725i injection valve (IDEX Corporation, Rohnert Park, CA, USA); the mobile phase was degassed by a solvent degasser mod. Degasys DG-1210 (Uniflows Co., Ltd., Tokyo, Japan). Chromatographic analyses were performed on an Inert Clone ODS column (150 × 4.6 mm, 5 μm, 100 Å, Phenomenex, Castel Maggiore, Italy) with a mobile phase of (A) water 20% and (B) acetonitrile 80% using an isocratic method. The flow rate was 1 mL/min, and the column temperature was 30 °C. The injection volume was 10 μL and the column eluates were monitored at 210 nm. Under these experimental conditions, the retention time of PEA was 8.8 ± 0.9 min.

### 3.6. Freeze-Dry Powder Characterization

PEA-LNP and conventional LNP (C-LNPs) suspensions were pre-frozen under different conditions and freeze-dried for 48 h (Lyovac GT2, Leybold-Heraues GmbH, Koln, Germany). The pre-frozen conditions adopted were: (i) the use of a cryoprotectant, mannitol, at two different concentrations, expressed as weight ratio between lipid matrix and cryoprotectant, 1:1 and 1:2, and without mannitol; (ii) the use of a pre-freezing temperature of −70 °C in a dry ice/acetone bath; and (iii) dilution with deionized water at 1:55 (expressed in volume ratio between particle suspension and water). These conditions were chosen on the basis of previous studies carried out through Design of Experiments analysis [21].

#### 3.6.1. Density

The bulk densities of freeze-dried PEA-LNPs and C-LNPs (1:1 and 1:2 ratio between lipids and mannitol, and without mannitol) were determined by pouring a known mass of powder (100 mg) under gravity into a graduated cylinder and recording the volume occupied by the powder, including the contribution of the inter-particulate void volume. The tapped densities of the samples were determined by measuring the volume of tapped mass until no further change in the powder, after about 150 taps, was observed, according to European Pharmacopoeia [48].

#### 3.6.2. Flowability

The density data were used to determine Carr’s Index and Hausner ratio, according to the following equations:

(1) $$\mathrm{Carr}’s\;\mathrm{Index}=\left(\frac{\rho_{tapped}-\rho_{bulk}}{\rho_{tapped}}\right)\times 100$$

(2) $$\mathrm{Hausner}\;\mathrm{ratio}=\frac{\rho_{tapped}}{\rho_{bulk}}$$

Carr’s Index (or Compressibility Index) is a measure of powder bridge strength and stability, and the Hausner ratio is a measure of the inter-particulate friction.

Regarding the angle of repose, the same PEA-LNP and C-LNP powders were allowed to flow freely through a funnel, maintained 4 cm above the bench surface, onto the center of a Petri dish (European Pharmacopoeia, 2021b). When the powder (200 mg) reached the side of the Petri dish, the height of the cylindrical cone was determined. From the Petri dish radius (*r*, cm) and cone height (*h*, cm), the angle of repose α was calculated using the following equation:

(3) $$tan\;\alpha =\frac{h}{r}$$

All of the measurements were performed in triplicate and the data were compared to the scale of flowability reported in the European Pharmacopoeia.

#### 3.6.3. In Vitro Respirability

In vitro respirability of freeze-dried PEA-LNPs, given different ratios with mannitol, were screened using a twin glass impinger (TGI) (Disa, Milan, Italy) following the procedure detailed in the European Pharmacopoeia [32]. The samples were loaded (about 30 mg), equally distributed, into three capsules (size 3, V-Caps Capsugel, Morristown, NJ, USA) and aerosolized using an RS01 device (Plastiape, Lecco, Italy) with a flow rate of 55 L/min, capable of producing a pressure drop of 4 kPa over the inhaler. The vacuum was applied for 5 s to obtain 5 L of air through the instrument during the experiment. After simulation, the TGI was disassembled and drug traces within the capsule/device (stage D), as well as in all the other stages, were collected with ethanol. Considering PEA solubility, 7 and 30 mL ethanol was inserted into the two TGI stages (stages A and B). The ethanol solutions obtained were sonicated at 40 °C with an ultrasonic bath for 15 min (Ultrasonic Cleaner USC-TH, VWR International Srl, Milano, Italy), filtered with 0.2 μm PTFE filters, and then analyzed using HPLC for the quantitative determination of PEA, using the same method described above. As reported in the European Pharmacopoeia, the experiment is considered valid if its yield (given by stage D, stage A and, stage B) falls within the range of 75 and 125% [32]. The determination of the amount of PEA deposited in the impinger allowed the calculation of deposition parameters: emitted dose (ED), as the amount of particle entirely released from the device, fine particle fraction (FPF), as the quantity of particles which accumulated in the lower part of the impactor, corresponding to the carrier with size <5 μm, and large particle fraction (LPF), which corresponds to those particles with size >5 μm.

### 3.7. Studies on Macrophage MH-S Cell Line

Murine alveolar macrophages from the MH-S cell line were employed in order to evaluate the cytotoxicity and cell internalization capacity of PEA-LNPs. MH-S cells were cultured under mixed conditions in RPMI 1640 medium supplemented with 2 mM L-glutamine, penicillin 100 UI/mL, 100 μg/mL streptomycin, and 10% FBS in T75 flasks at 37 °C and 5% CO_2_.

#### 3.7.1. Cytotoxicity by MTT Test

Cells were seeded at a density of 120,000 cells/well in a 24-well plate in complete medium. Cells were then incubated for 6 h with freeze-dried PEA-LNPs and C-LNPs at three different concentrations: 0.16, 0.32, and 0.64 mg/mL. After the incubation time, 100 μL of a 5 mg/mL MTT solution was added to each well. After 1 h of incubation, the medium was removed, and 1 mL of dimethyl sulfoxide (DMSO) was added to dissolve blue formazan crystals in each well. MTT conversion to formazan by metabolically viable cells was monitored using a multiplate reader (TecanGenios Pro with Magellan 6 software, MTX Lab Systems, Bradenton, FL, USA) at an optical density of 535 nm. The MTT test was performed in triplicate, and cell viability was expressed as a percentage of cell survival compared with untreated cells. The experiments were performed in triplicate.

#### 3.7.2. Preparation of Coumarin-6 Labelled PEA-LNPs

Labelled PEA was obtained by adding coumarin-6 (0.1%, *w*/*w*) to an ethanol solution of a known weight of PEA. Ethanol was removed in a vacuum concentrator and the resulting powder was stored at 25 °C in the dark. The coumarin-6 labelled PEA was used to prepare labelled PEA-LNPs.

Coumarin-6 in vitro release from labelled PEA-LNPs was evaluated over the course of 24 h. Labelled particles (40 mg) were incubated at 37 °C in 40 mL of phosphate buffer (20 mM, pH 7.4) or RPMI 1640 without phenol red, under magnetic stirring. One milliliter of suspension was withdrawn from the system at time intervals of 30 min and replaced with 1 mL of fresh solvent to maintain constant volume. The sample was subjected to centrifugation at 13,000× *g* using a Microcon^®^ centrifugal filter (100 kDa, Millipore Corporation, Bedford, MA, USA), and coumarin-6 content was determined in the supernatant by vis-spectroscopy at 459 nm. The analysis was performed in triplicate.

#### 3.7.3. Flow Cytometry

For the flow cytometry analysis, the cells were seeded at a density of 500,000 cells/well in a six-well plate and incubated at 37 °C with the sample suspensions stirred using a vortex for 1 min. Untreated cells and unlabeled C-LNPs were used as controls. The ability of coumarin-6 labelled PEA-LNPs to be taken up by MH-S cells was evaluated at 1 h and 3 h of incubation, at a concentration of 0.32 mg/mL. After each period of incubation, cells were washed twice with PBS and detached using trypsin; then, the cells were collected with PBS and centrifuged at 2500 rpm for 5 min at 25 °C. Subsequently, the pellet was suspended in PBS and analyzed with a flow cytometer (Attune NxT, ThermoFisher Scientific, Monza, Italy) with a 525 nm argon laser. Each test was carried out in duplicate.

#### 3.7.4. Confocal Microscopy

For the internalization study, MH-S cells were seeded at a density of 150,000 cells/plate in glass bottom dishes with a diameter of 22 mm. After 24 h, cultured cells were incubated with labelled PEA-LNPs (0.32 mg/mL) for 1, 3, and 6 h, then immediately fixed with paraformaldehyde (3%, *w*/*v*) for 20 min at room temperature and washed in PBS three times. Then, the cell nuclei were stained with 250 μL of Hoechst 33,342 stain (blue, 2 μg/mL in PBS) for 20 min at room temperature. Untreated cells were used as the control. The cells were observed under a filter set for yellow fluorescence (exciting wavelength of 457 nm, emission wavelength of 501 nm). Images were acquired using a 63X objective on a Leica SP8 AOBS system (Leica Microsystems, Wetzlar, Germany), equipped with a WLL.

### 3.8. Statistical Analysis

Data obtained were evaluated statistically using one-way analysis of variance (ANOVA). Significance was indicated by *p* < 0.05 (* *p* ≤ 0.05; ** *p* < 0.02; *** *p* < 0.01).

## 4. Conclusions

This report is focused on the development of an inhalable nano-drug delivery system designed to efficiently and safely transport therapeutic agents in the airways. Through the investigation of PEA-LNPs, suitability for inhalation administration and favorable respirability attributes were attained in freeze-dried PEA-LNP powder. The evaluation of the powder cytotoxicity on alveolar macrophages revealed, even at elevated concentrations, non-toxic characteristics. The non-toxic nature of PEA-LNPs is significant for ensuring their safety profile in therapeutic applications. Furthermore, we observed a rapid dissolution of PEA and swift uptake by macrophages within just one hour of incubation, highlighting the potential for rapid onset of therapeutic action in targeted lung tissues. The favorable respirability attributes observed in the freeze-dried powder of PEA-LNPs suggest promising potential for efficient pulmonary delivery. These findings underscore the importance of further investigations for exploring PEA-LNPs as a viable option for inhalation therapy. Future perspectives will involve the formulation and optimization of drug loaded PEA-LNPs, and the improvement of macrophage targeting by decorating the particle surface with specific molecules.

## Figures and Tables

**Table 1:** Bulk density, tap density, Housner ratio, Carr’s Index, and angle of repose for the different samples tested. The data are presented as the average ± SD (*n* = 3).

Sample (Lipids/Mannitol Ratio)	Bulk Density (g/cm^3^) ± SD	Tap Density (g/cm^3^) ± SD	Housner Ratio	Carr’s Index (%)	Angle of Repose (°) ± SD
C-LNPs (no mannitol)	0.07 ± 0.02	0.08 ± 0.01	1.14	12.5	34.1 ± 1.7
PEA-LNPs (no mannitol)	0.11 ± 0.01	0.16 ± 0.03	1.45	31	46.7 ± 2.7
PEA-LNPs (1:1 ratio)	0.04 ± 0.01	0.05 ± 0.02	1.25	20	35.6 ± 4.2
PEA-LNPs (1:2 ratio)	0.05 ± 0.01	0.05 ± 0.01	1.08	7	27.0 ± 4.8

**Table 2:** Emitted dose (ED), fine particle fraction (FPF), and large particle fraction (LPF) values for the different samples used for the in vitro respirability test.

Sample (Lipids/Mannitol Ratio)	ED (%) ± SD	FPF (%) ± SD	LPF (%) ± SD
PEA-LNPs (no mannitol)	61.9 ± 3.9	0.1 ± 0.0	61.8 ± 2.8
PEA-LNPs (1:1 ratio)	81.5 ± 2.5	9.6 ± 3.2	71.9 ± 2.3
PEA-LNPs (1:2 ratio)	84.1 ± 1.0	16.4 ± 1.7	67.7 ± 0.8

**Table 3:** Mean percentage of positive MH-S cells, with respect to the total cell population, according to flow cytometer analysis after 1 and 3 h of treatment.

Sample	1 h	3 h
	Fluorescence (%)	
Untreated cells	1.03 ± 0.09	0.49 ± 0.16
Not labelled C-LNPs	0.54 ± 0.18	0.43 ± 0.02
Labelled PEA-LNPs	94.65 ± 0.67	96.73 ± 0.81

## Data Availability

Data are contained within the article and Supplementary Materials.

## Supplementary Materials

The following supporting information can be downloaded at: https://www.mdpi.com/article/10.3390/molecules29071616/s1, Table S1: Simulated Lung Fluid (SLF) at pH 7.4 salt composition; Table S2: Summary of preliminary samples prepared with different ratio between the lipid components. Size and polydispersity index (PDI).
