# Supplementary material for: Inflammatory-Targeted Lipid Carrier as a New Nanomaterial to Formulate an Inhaled Drug Delivery System
Source: Molecules. 2024 Apr 3;29(7):1616. doi: 10.3390/molecules29071616 (PMC11013801; doi:10.3390/molecules29071616)
Supplement: Supplementary file 1 [file molecules-29-01616-s001.zip › molecules-2903172-supplementary.pdf]

Supplementary material

Inflammatory-targeted lipid carrier as a new nanomaterial to formulate an inhaled drug delivery system

Eleonora Maretti, Federica Gioia, Cecilia Rustichelli, Susanna Molinari and Eliana Leo*

Department of Life Sciences, University of Modena and Reggio Emilia, via G. Campi 103, 41125, Modena, Italy

**corresponding author: Eliana Leo, Department of Life Sciences, Via Campi, 103, 41125 Modena, Italy, tel *39 59 2055885; email: eliana.leo@unimore.it*

Table S1. Summary of preliminary samples prepared with different ratio between the lipid components. Size and polydispersity index (PDI)

PEA, stearic acid and cholesteryl stearate (weight ratio)	Size (nm ± SD)	PDI
1 : 0.5 : 0.5	568 ± 43	0.524 ± 0.143
1 : 1 : 1	430 ± 20	0.362 ± 0.015
1 : 2 : 1	388 ± 1	0.306 ± 0.052
1 : 2 : 2	305 ± 11	0.285 ± 0.043
1 : 2.6 : 2	253 ± 15	0.185 ± 0.100

Table S2. Simulated Lung Fluid (SLF) at pH 7.4 salt composition.

Components	g/l
Magnesium chloride hexahydrate	0.2033
Sodium chloride	6.0193
Potassium chloride	0.2982
Sodium sulphate anhydrous	0.071
Calcium chloride dihydrate	0.3676
Sodium acetate trihydrate	0.9526
Sodium hydrogen carbonate	2.6043
Sodium citrate dihydrate	0.097
Sodium phosphate monobasic monohydrate	0.142
